# Analgesic Efficacy of EMLA Cream Among Patients Undergoing Hemorrhoidectomy: A Systematic Review and Meta-Analysis of Randomized Controlled Trials

**DOI:** 10.7759/cureus.66423

**Published:** 2024-08-08

**Authors:** Kalthoum AlAwadhi, Fahad A Allafi, Bader A Almukaimi, Ahmad Alkandari, Saoud A Alenezi, Awatef Alenezi, Shaikhah Alenezi, Sara Alenezi, Nasser Alenezi, Abdullah Fahiman, Abdulwahab Alsalem, Muteb Alotaibi

**Affiliations:** 1 Medicine and Surgery, Kuwait Institute for Medical Specializations, Kuwait City, KWT; 2 General Surgery, Jahra Hospital, Jahra, KWT; 3 Medicine, Alexandria University, Alexandria, EGY; 4 Surgery, Amiri Hospital, Kuwait City, KWT; 5 Medicine and Surgery, King Faisal Specialist Hospital and Research Centre, Riyadh, SAU

**Keywords:** opioid alternative, meta-analysis, pain, emla, hemorrhoidectomy

## Abstract

Post-hemorrhoidectomy pain is a concerning complication for patients and doctors, mainly due to perianal skin dissection and the complex innervation of the area. Therefore, our aim is to explore the analgesic efficacy and safety of EMLA cream among patients undergoing hemorrhoidectomy. We conducted a comprehensive search of five electronic databases (PubMed, Scopus, Web of Science, Embase, Cochrane) from inception until July 6, 2024. A risk of bias assessment was performed using the Risk of Bias Version 2 of the Cochrane risk-of-bias tool for randomized trials (RoB-2). Only randomized controlled trials (RCTs) were included. Our outcomes of interest were pain assessment using the Visual Analogue Scale (VAS) score, patient satisfaction, meperidine dosage, frequency of meperidine requests, and single urinary catheterization. We used RevMan software to conduct the statistical analysis. Dichotomous data were pooled as relative risk (RR), while continuous data were pooled as mean difference (MD). Four RCTs were included in our review. Two RCTs showed a low overall risk of bias, while one RCT showed a high risk, and the last one showed some concerns. Our analysis showed a significant difference between the two groups, favoring the EMLA group over the control group, upon arrival at the recovery room and at night on the day of the operation (MD=-1.76, 95% CI (-3.17, -0.36), p=0.01 and MD=-1.65, 95% CI (-2.48, -0.81), p=0.0001, respectively). However, there was no significant difference between the two groups in the morning on the day after the operation (MD=-0.9, 95% CI (-2.02, 0.21), p=0.11). Moreover, patients who used EMLA cream reported increased patient satisfaction compared to those who did not. However, there was no significant difference between both groups in terms of the use of urinary catheterizations. In conclusion, our study showed that applying EMLA cream over the perianal area after hemorrhoidectomy can effectively reduce postoperative pain and decrease the required opioid dosage and patient requests. This ultimately leads to increased patient satisfaction. However, further studies are still required to confirm these findings.

## Introduction and background

Hemorrhoids are the most widespread anorectal disease, with a global incidence of 4-36%, peaking at 45-65 years of age [1]. The sex ratio is approximately 1:4, with a female prevalence [1]. Post-hemorrhoidectomy pain is a concerning complication for patients and doctors, mainly due to perianal skin dissection and the complex innervation of the area. It has also been suggested that pain arises from spasms of the internal anal sphincter [2]. Pain can prolong hospitalization, increase costs, and lead to complications for the patient [3,4]. When patients undergo hemorrhoidectomy, it is expected that they will experience moderate to severe pain during the first two weeks after surgery [5,6]. Therefore, it is crucial to provide adequate and accessible analgesia for patients during both the immediate postoperative stage and after discharge. Furthermore, effective analgesia can also resolve postoperative urinary retention caused by severe perianal pain [7]. This not only increases patient satisfaction but also improves overall outcomes and subsequently reduces readmissions [8,9].

Pain management postoperatively traditionally consists of local anesthetics (i.e., bupivacaine or ropivacaine) infiltrated into the wound [10,11]. Topical ointments such as 10% metronidazole or 10% sucralfate and oral metronidazole are also used to reduce post-hemorrhoidectomy pain [12]. Although local injectable anesthetics reduce immediate pain for up to six hours after surgery, they cannot be used for long-term pain management because of their invasive nature [13]. Local anesthetic infiltration was also proven ineffective in reducing the need for postoperative pain management; it was shown that patients who received bupivacaine did not need less analgesia compared to those who did not receive any injectable anesthetic [14]. The presence of moderate to severe pain in up to 65% of patients receiving local anesthetic infiltration postoperatively further solidifies the need for locally applied analgesia as an adjunct to the perianal infiltration [15].

A suggested alternative is the use of a eutectic mixture of topical anesthetics (EMLA) which consists of prilocaine and lidocaine. This mixture has been consistently proven to be effective and safe in relieving pain in various situations such as sharp wound debridement, post-circumcision, repair of perineal trauma, and analgesia for thyroid fine needle aspiration [16-19]. The use of other topical ointments (metronidazole 10%, sucralfate 10%) for postoperative pain has paved the way for EMLA cream to be considered as a potentially safe and effective alternative following hemorrhoidectomy. EMLA cream has a rapid onset of action and is easy to apply, making it a favorable choice for patients. However, given the absence of a definitive consensus on this subject matter, the primary objective of this meta-analysis is to investigate the efficacy and safety of EMLA cream in the management of post-hemorrhoidectomy pain.

## Review

Methods and materials

We conducted a systematic review and meta-analysis following the guidelines of the Preferred Reporting Items for Systematic Reviews and Meta-Analyses (PRISMA) statement [20] and the Cochrane Handbook for Systematic Reviews of Interventions [21]. We performed a comprehensive search of five electronic databases (PubMed, Scopus, Web of Science, Embase, Cochrane) from inception until July 6, 2024. The detailed search strategy can be seen in Table 1. We used the following search strategy without filters: (hemorrhoidectomy OR Hemorrhoidectomies) AND (EMLA OR lidocaine-prilocaine OR Lidocaine Prilocaine OR Eutectic OR Oraqix OR PSD 502 OR PSD502 OR PSD-502 OR Fortacin). Furthermore, we conducted a manual search through Google Scholar, ResearchGate, and references to avoid missing any relevant studies. No specific filter based on country or language was applied during the systematic search. 

**Table 1:** The exact literature search strategy used in every database.

Database	Search strategy
		PubMed	All Fields: (hemorrhoidectomy OR Hemorrhoidectomies) AND (EMLA OR lidocaine-prilocaine OR Lidocaine Prilocaine OR Eutectic OR Oraqix OR PSD 502 OR PSD502 OR PSD-502 OR Fortacin)
Scopus	Article title, Abstract, Keywords: (hemorrhoidectomy OR Hemorrhoidectomies) AND (EMLA OR lidocaine-prilocaine OR Lidocaine Prilocaine OR Eutectic OR Oraqix OR PSD 502 OR PSD502 OR PSD-502 OR Fortacin)
Web of Science	All Fields: (hemorrhoidectomy OR Hemorrhoidectomies) AND (EMLA OR lidocaine-prilocaine OR Lidocaine Prilocaine OR Eutectic OR Oraqix OR PSD 502 OR PSD502 OR PSD-502 OR Fortacin)
Cochrane Central Register of Controlled Trials (CENTRAL)	Title Abstract Keyword: (hemorrhoidectomy OR Hemorrhoidectomies) AND (EMLA OR lidocaine-prilocaine OR Lidocaine Prilocaine OR Eutectic OR Oraqix OR PSD 502 OR PSD502 OR PSD-502 OR Fortacin)
Embase	All Fields: (hemorrhoidectomy OR Hemorrhoidectomies) AND (EMLA OR lidocaine-prilocaine OR Lidocaine Prilocaine OR Eutectic OR Oraqix OR PSD 502 OR PSD502 OR PSD-502 OR Fortacin)

Eligibility Criteria

We included the clinical trials that met the following eligibility criteria (PICOS): (1) population: patients with grade III or IV hemorrhoids who underwent hemorrhoidectomy, (2) intervention: EMLA cream, regardless of the dose and application method, (3) control: no treatment or placebo, including non-analgesic creams such as moisturizing cream, petrolatum ointment, or neomycin, (4) outcomes: post-hemorrhoidectomy pain assessed using the Visual Analogue Scale (VAS) score, patient satisfaction, analgesia dosage (mg), frequency of analgesic requests, and single urinary catheterization, and (5) study design: we included only randomized controlled trials (RCTs). We excluded review articles, case reports, observational studies, studies that were not published in the English language, studies whose complete full texts were not available, studies that were reported as abstracts only, and studies whose data were not reliable for extraction and analysis.

Study Selection and Data Extraction

After extracting the eligible studies from the databases, two independent authors conducted the first (title and abstract) and second (full-text) scans. They also extracted the data of interest, which included the characteristics of the studies (e.g., study design, country, number of participants), the characteristics of the participants (age, gender, and body weight), and the outcomes (e.g., pain assessment, patient satisfaction, meperidine dosage). This data was based on a previously prepared sheet. When the standard deviation (SD) was not reported, the SD of the most relevant study was used, following Cochrane guidelines [21]. Any disagreements were resolved through discussion.

Risk of Bias

Two reviewers separately assessed the included studies using the Risk of Bias Version 2 of the Cochrane risk-of-bias tool for randomized trials (RoB-2) [22]. The Cochrane tool for assessing the possibility of bias domains included in RoB-2 covers all types of bias that are currently understood to affect the results of randomized trials. These are as follows: (D1) bias arising from the randomization process, (D2) bias due to deviations from intended interventions, (D3) bias due to missing outcome data, (D4) bias in the measurement of the outcome, and (D5) bias in the selection of the reported result. The risk-of-bias judgment for each domain was determined by one of these levels: "Low risk of bias," "Some concerns," or "High risk of bias." These judgments were based on RoB-2 signaling questions provided by the guidance.

Statistical Analysis

We used RevMan software (version 5.3 for Windows) to conduct the statistical analysis, employing a random-effects model [23]. Dichotomous data were pooled as relative risk (RR) using the Mantel-Haenszel (M-H) method, while continuous data were pooled as mean difference (MD) using the inverse variance (IV) method, both with a 95% confidence interval (CI). We reported p-values that are less than 0.05 as statistically significant as it is small enough to reject the null hypothesis. Statistical heterogeneity of the included studies was assessed by the chi-squared test (Cochrane Q test) and the I-square test. Significant heterogeneity was reflected by a p-value less than 0.1 for the chi-squared test and I-square test >50%. We conducted a subgroup analysis for pain assessment according to the time of assessment (at the arrival recovery room, at night on the operation day, and at the morning on the day after the operation). Publication bias assessment was not done as the number of included studies is less than 10 [21].

Results

Search Outcomes and Selection of Studies

**Figure 1:**
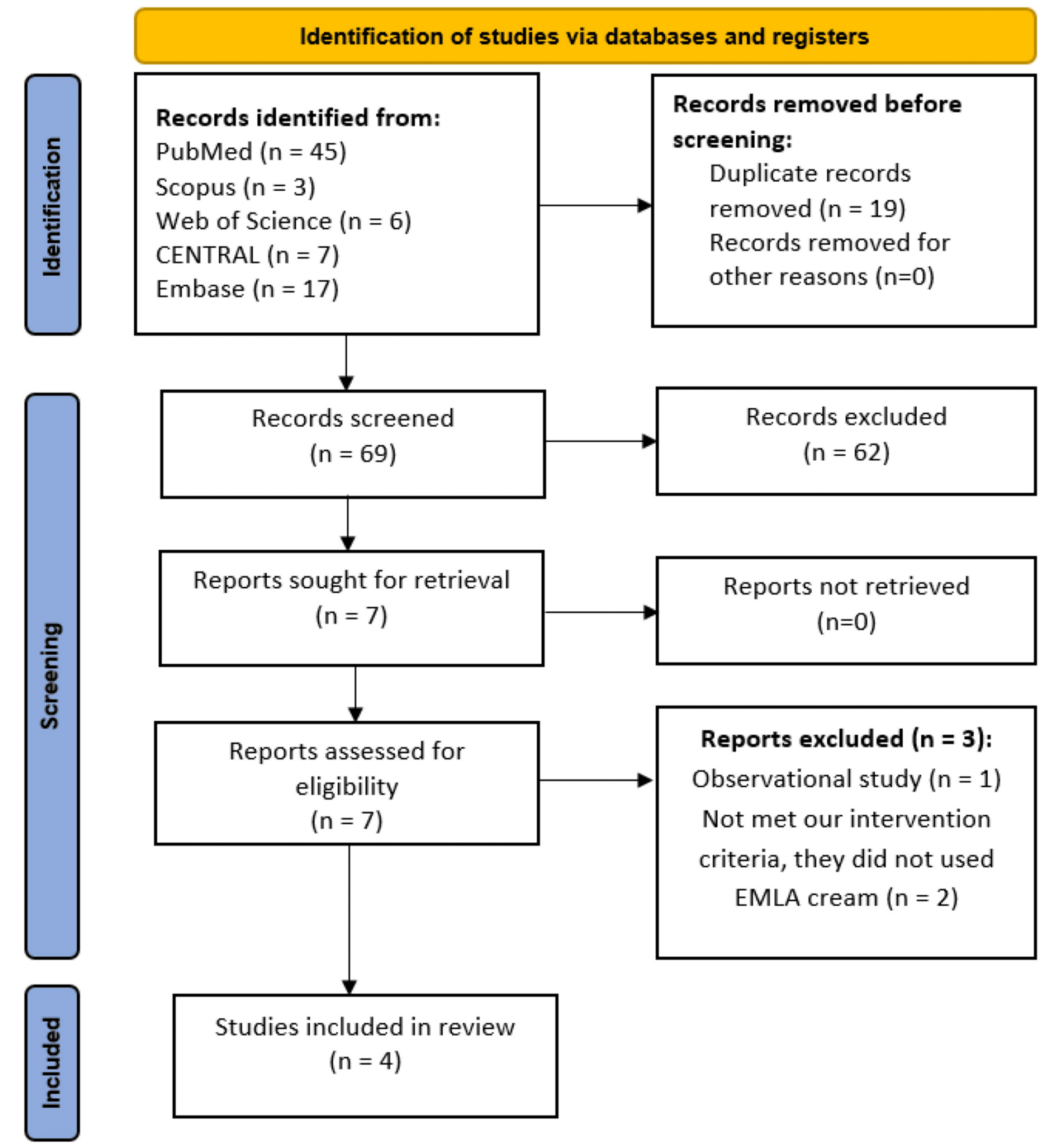
PRISMA flow diagram of studies' screening and selection. PRISMA: Preferred Reporting Items for Systematic Reviews and Meta-Analyses

Our systematic search initially found 78 records. Nineteen studies were excluded as duplicate studies. The remaining 69 citations underwent title and abstract screening and were reduced to seven studies. Finally, four RCTs were included in the meta-analysis [24-27]. More details are shown in Figure 1.

Characteristics of Included Studies

The number of patients who were included in the meta-analysis was 248, including 124 patients treated with 5 grams of EMLA cream and 124 patients treated with non-analgesic creams like 5 grams of moisturizing cream, petrolatum ointment, or neomycin. The duration of follow-up was 24 hours and the pain assessment tool was VAS score in almost all studies. A summary of the eligible studies and the characteristics of their participants are presented in Table 2 and Table 3, respectively.

**Table 2:** Summary of the included studies. References: [24-27]

Study ID	Study design	Country	Number of patients	Intervention	Control group	Main inclusion criteria	Follow-up period
Rahimi et al. 2012 [24]	Randomized controlled clinical trial	Iran	N=60	5 grams of EMLA cream	5 grams of petrolatum ointment	Patients with grade III or IV hemorrhoids	24 hours
Roxas et al. 2003 [25]	Randomized controlled clinical trial	Philippines	N=98	5 grams of EMLA 5% cream	5 grams of moisturizing cream	Symptomatic patients with grade III or IV external, internal, and/or mixed hemorrhoids	Not reported
Shiau et al. 2007 [26]	Randomized controlled clinical trial	Taiwan	N=60	5 grams of EMLA cream	5 grams of neomycin	Patients with grade III or IV hemorrhoids	24 hours
Shiau et al. 2008 [27]	Randomized controlled clinical trial	Taiwan	N=30	5 grams of EMLA cream	5 grams of neomycin	Patients with grade III or IV hemorrhoids	24 hours

**Table 3:** Baseline characteristics of the participants in the included studies. NR: not reported; SD: standard deviation; VAS: Visual Analogue Scale References: [24-27]

Study ID	Age (years), mean (SD)		Gender, n (male/female)		Body weight (kg), mean (SD)		Pain assessment tool
	EMLA group	Control group	EMLA group	Control group	EMLA group	Control group	
Rahimi et al. 2012 [24]	39.33 (15.24)	35.53 (12.51)	11/19	19/11	NR	NR	NR
Roxas et al. 2003 [25]	33.49	36.96	29/20	27/22	NR	NR	VAS score
Shiau et al. 2007 [26]	43.93 (12.92)	43.43 (11.65)	14/16	11/19	58.73 (9.65)	60.10 (10.02)	VAS score
Shiau et al. 2008 [27]	41 (10)	44 (12)	7/8	6/9	58 (8)	54 (6)	VAS score

Pain (VAS)

**Figure 2:**
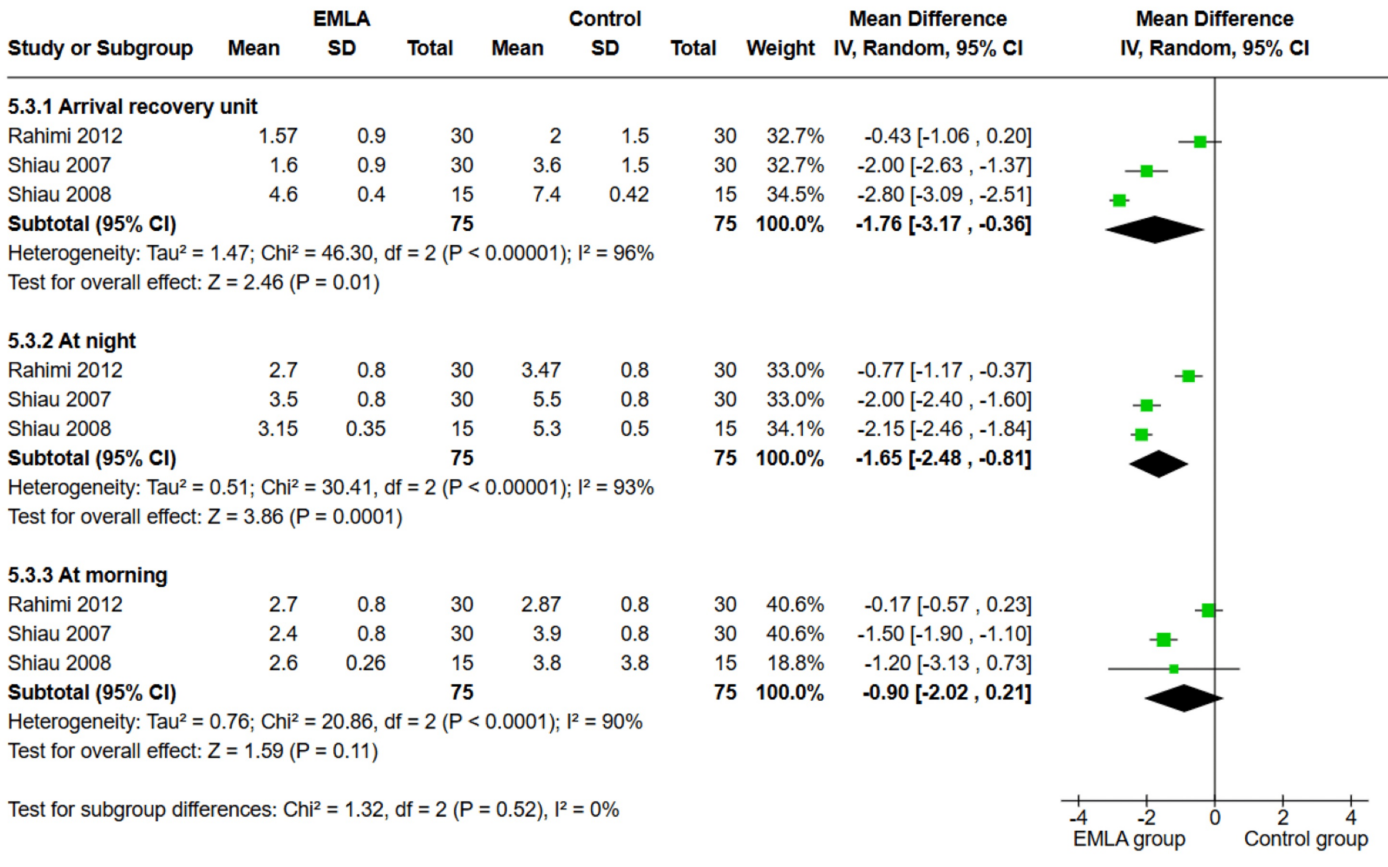
Forest plot of MD of VAS score with 95% confidence interval. MD: mean difference; VAS: Visual Analogue Scale References: [24,26,27]

Three studies measured pain using the VAS at different time points during the study (n=150 participants). Our analysis showed a significant difference between the two groups, favoring the EMLA group over the control group, upon arrival at the recovery room and at night on the day of the operation (MD=-1.76, 95% CI (-3.17, -0.36), p=0.01 and MD=-1.65, 95% CI (-2.48, -0.81), p=0.0001, respectively). However, there was no significant difference between the two groups in the morning on the day after the operation (MD=-0.9, 95% CI (-2.02, 0.21), p=0.11). The analysis showed heterogeneity in the results at all-time points (p<0.1, I^2^>90%), as shown in Figure 2.

Meperidine Dosage (mg)

**Figure 3:**
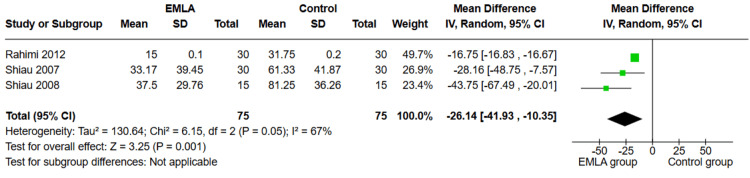
Forest plot of MD of meperidine dosage with 95% confidence interval. MD: mean difference References: [24,26,27]

Three studies investigated the meperidine dosage (n=150 participants). The overall MD showed that there was a significant difference between the two groups favoring the EMLA group (MD=-26.14, 95% CI (-41.93, -10.35); p=0.001) as shown in Figure 3. The pooled studies were heterogeneous (p=0.05; I²=67%).

Frequency of Meperidine Requests

**Figure 4:**
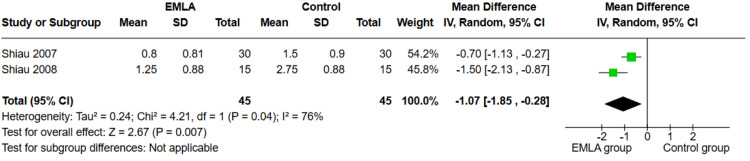
Forest plot of MD of frequency of meperidine requests with 95% confidence interval. MD: mean difference References: [26,27]

Two studies report this outcome (n=90). Our analysis showed that the EMLA group required a significantly lower frequency of meperidine requests compared to the control group (MD=-1.07, 95% CI (-1.85, -0.28); p=0.007) as shown in Figure 4. The pooled analysis was heterogeneous (p=0.04; I²=76%).

Patient Satisfaction (5-Point)

**Figure 5:**
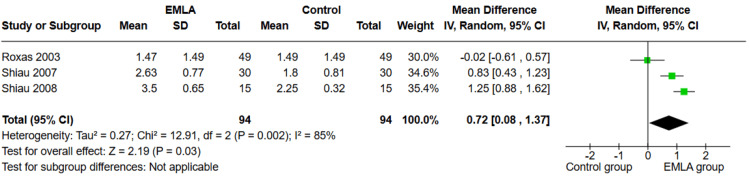
Forest plot of MD of patient satisfaction with 95% confidence interval. MD: mean difference References: [25,26,27]

Three studies reported this outcome (n=188). Our analysis showed that patients who received EMLA had higher satisfaction rates compared to the control group (MD=0.72, 95% CI (0.08, 1.37); p=0.03), as shown in Figure 5. The pooled studies were heterogeneous (p=0.002; I²=85%).

Single Urinary Catheterization

**Figure 6:**
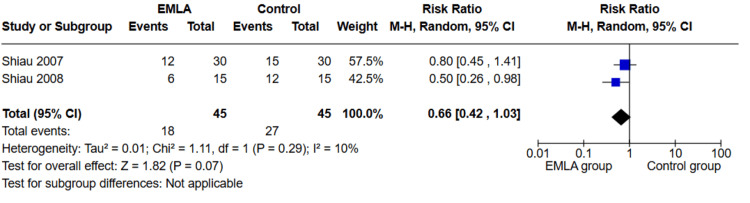
Forest plot of RR of single urinary catheterization with 95% confidence interval. RR: risk ratio References: [26,27]

Two studies investigated single urinary catheterization (n=90 participants). The overall RR showed no significant difference between the two groups (RR=0.66, 95% CI (0.42, 1.03); p=0.07), as shown in Figure 6. The pooled studies were homogeneous (p=0.29; I²=10%).

Risk of Bias

**Figure 7:**
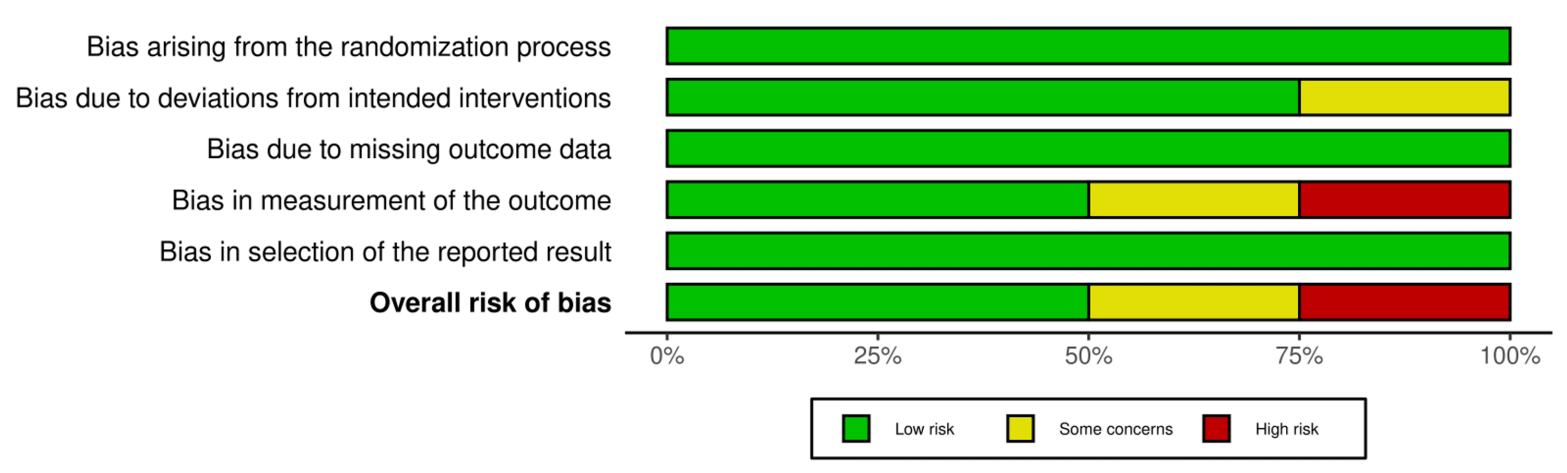
The risk of bias summary graph.

Out of the four randomized clinical trials, two studies [24,27] showed a low risk of overall bias. Roxas et al. [25] showed a high risk of bias regarding the measurement of outcomes as there was no assessor blinding. However, Shiau et al. [26] had some concerns in D2 and D4 due to insufficient information to assess the validity of each domain (Figure 7).

Discussion

This first-ever meta-analysis of RCTs found that patients using EMLA cream required lower meperidine dosages (in terms of quantity) and fewer meperidine requests (in terms of frequency) compared to the control group. Patients' VAS scores, measured at three different timeframes (upon arrival at the recovery unit, at night, and the next morning), were significantly lower in the EMLA group compared to the control group, except in the next morning, where there was no significant difference between the EMLA and control groups. Moreover, patients who used EMLA cream reported increased patient satisfaction compared to those who did not. However, there was no significant difference between both groups in terms of the use of urine catheterizations.

EMLA, or a eutectic mixture of local anesthetic, is created as an oil-water emulsion containing 2.5% lidocaine and 2.5% prilocaine. The penetration of this substance is remarkable and has been used topically on intact skin, particularly in pediatrics. It has been utilized in various procedures, such as venipuncture [28] and post-circumcision [17]. Its use in surgical procedures may require further evaluation for toxicity, as it can be more readily absorbed systemically and result in higher plasma levels when applied to diseased skin [29]. However, a study conducted using high doses (5-10 g) of EMLA cream on painful leg ulcers for 24 hours found that combined EMLA (lidocaine-prilocaine) levels did not exceed one-fifth of the toxicity threshold [30]. Blanke and Hallernn further established its safety by using EMLA cream on over 1000 patients with leg ulcers, administering doses ranging from 3 g to 150 g, with no reported neurological complications [16]. Methemoglobinemia is a known adverse event associated with prilocaine use, but it is rarely reported unless it is related to drug-drug interactions [31,32] or inappropriately high doses are applied to non-intact skin [32,33]. Further studies are still recommended to evaluate EMLA toxicity when applied to perianal skin and mucosa, as these areas may have higher absorption rates.

There are non-opioid analgesics, parecoxib, acetaminophen, and EMLA, with proven effectiveness following various surgical procedures [19,34]. The widespread use of non-opioid analgesics can help reduce opioid-induced side effects [19,34]. EMLA was found to decrease the frequency of requests for additional meperidine doses postoperatively. The difference between the two groups demonstrated increased pain tolerance when patients used EMLA cream in combination with meperidine injections compared to patients who used meperidine alone. Administering meperidine less frequently reduces costs, decreases the workload of healthcare workers, and helps reduce the risk of opioid misuse. Reducing the amount of opioids patients receive can be an important step towards reducing long-term opioid dependence [35].

VAS is a graded score with a range from 0 (pain-free) to 10 (worst pain they have ever felt). It is used to assess the severity of postoperative pain. The VAS score was analyzed upon arrival to the recovery unit, at night, and the next morning. The patients using EMLA cream had significantly lower VAS scores upon arrival to the recovery unit and at night. However, no significant difference was found between the two groups when assessing the VAS score the next morning. This may be due to the administration of opioid analgesics to reduce pain levels, which could not be delayed for ethical reasons [26,27]. The pain-relieving properties of EMLA cream can be attributed to its ease of use, and the persistent decrease in VAS score may be attributed to patients repeatedly applying the cream.

Patient satisfaction was higher in patients who used the EMLA cream compared to the control group. This may indicate a direct relationship between postoperative pain relief and patient satisfaction. Another factor contributing to the satisfaction of patients using the EMLA cream is the ease of use. The cream is applied topically by the patient, providing pain relief without the need for anesthetic injections. This helps patients resume their daily activities without the burden of significant pain or discomfort.

Postoperative urinary retention is multifactorial. Restriction of perioperative fluid, avoidance of spinal anesthesia, and avoiding anal packing are some recommendations to reduce urinary retention [27]. However, pain is a significant cause of postoperative urinary retention, especially with anorectal surgery [7]. There was no significant difference between the patients who took EMLA cream and those in the control group. However, this does not diminish the role of pain control in urinary retention. Its correlation is not well represented as we only included two studies that reported this outcome; additionally, the p-value was borderline. Accordingly, further studies could be useful to establish more accurate and reliable results.

This is the first meta-analysis comparing the efficacy of EMLA cream with a control group. Although the study has reliable outcomes and methods, it has some limitations. Certain outcomes (meperidine dosage and VAS score at different timeframes) had heterogeneity which may affect the reliability of the study. Rahimi et al. may have caused the heterogeneity as they did not report any information about the technique used and whether gauze was used for coverage regarding the application of topical ointment [24]. However, other included studies described the technique for applying EMLA cream and how the wound was covered with gauze to maintain a closed area. Roxas et al. [25] may have caused heterogeneous results regarding patient satisfaction, and this may be due to the timing of the topical agent placement; the other included studies had the topical agent placed postoperatively [26,27], while Roxas et al. [25] had it placed for patients 45 minutes preoperatively. The difference in the non-analgesic topical creams used as a control group may have also contributed to the heterogeneity. Neomycin was used in Shiau et al. and Shiau et al., while petrolatum jelly was used in Rahimi et al. [24,26,27]. Neomycin could have indirect anti-inflammatory and analgesic effects via its bactericidal effects [36].

Lower VAS scores during the early postoperative period may be due to the lingering effects of spinal anesthesia. Further studies with a larger sample size are needed to generalize our conclusions to a wider population. Additionally, the exclusion of patients with preexisting kidney or liver diseases, as well as those with methemoglobinemia, makes it difficult to assess the drug's effect on these patients. Further studies should include these patient populations to establish optimal dosing, ensuring maximum efficacy and minimal side effects.

## Conclusions

Our study showed that applying EMLA cream over the perianal area after hemorrhoidectomy can effectively reduce postoperative pain and decrease the required opioid dosage and patient requests. This ultimately leads to increased patient satisfaction. However, further studies are still required to confirm these findings.
